# A CTC/DTC-centered temporal framework of bone metastasis: From dissemination to dormancy and reactivation

**DOI:** 10.1016/j.jbo.2026.100793

**Published:** 2026-08-04

**Authors:** Zijie Yuan, Hao Zhang, Jingyu Xing, Xinghao Cao, Genkun Liu, Tao Tan, Chenglong Zhao, Cheng Yang

**Affiliations:** a Department of Orthopedic Oncology, Changzheng Hospital, Naval Medical University, Shanghai 200003, China; b Department of Basic Medicine, Naval Medical University, Shanghai 200003, China; c Department of Orthopedics, The 905th Hospital of the People's Liberation Army Navy, Naval Medical University, Shanghai 200052, China.

**Keywords:** Bone metastasis, Circulating tumor cells, Disseminated tumor cells, Bone marrow niche, Metastatic dormancy, Minimal residual disease

## Abstract

Bone metastasis is a major cause of morbidity and poor survival in solid tumors, yet its progression is often recognized only after overt lesion formation. In this review, we propose a circulating tumor cell/disseminated tumor cell (CTC/DTC)-centered temporal framework that conceptualizes bone metastasis as a sequential and selection-driven process linking dissemination, bone-specific homing, DTC establishment, dormancy, and reactivation. We summarize the key cellular and molecular mechanisms governing these transitions, with particular emphasis on tumor cell plasticity, bone marrow niche interactions, and the dormancy–reactivation interface. We further discuss the translational relevance of this approach for risk stratification, minimal residual disease assessment, treatment monitoring, and emerging experimental strategies, including single-cell multi-omics, spatial omics and biomimetic bone microenvironment models. This perspective shifts the view of bone metastasis from a static, imaging-detectable lesion to a time-resolved biological process and highlights potential windows for earlier risk assessment, disease monitoring, and biology-guided intervention.

## Highlights

• Bone metastasis is reframed as a CTC/DTC-centered temporal process.
• CTC-to-DTC transition is shaped by immune selection and niche homing.
• Bone marrow niches regulate DTC dormancy, reactivation, and outgrowth.
• Candidate CTC/DTC markers may support relapse risk stratification.
• Single-cell and spatial omics can resolve DTC–niche interactions.

## 1 Background

Bone is one of the most frequent distant metastatic sites for a wide range of malignant tumors. Epidemiological studies indicate that bone metastases occur in approximately 73% of patients with breast cancer, 68% with prostate cancer, 42% with thyroid cancer, 36% with lung cancer, and 35% with renal cancer [1]. Bone metastases are commonly accompanied by skeletal-related events (SREs), including bone pain, hypercalcaemia, pathological fractures, and spinal cord compression, which substantially impair quality of life, increase healthcare burden, and are associated with advanced disease stages and poor survival outcomes [2], [3]. Recent real-world evidence from the OPTIMOS study further supports the persistent clinical burden of bone metastases and SREs in contemporary oncology practice, highlighting the need to optimize bone metastasis management even in the era of bone-targeting agents [4]. Because metastatic lesions in bone are often not amenable to complete surgical resection, their clinical management relies heavily on multidisciplinary collaboration and individualized therapeutic strategies, posing persistent challenges for cancer treatment [5].

At present, clinical detection of bone metastasis primarily depends on imaging modalities such as computed tomography (CT), magnetic resonance imaging (MRI), bone scintigraphy, and positron emission tomography–computed tomography (PET-CT) [6]. In most cases, these approaches enable diagnosis only after overt bone destruction or the formation of macroscopic metastatic lesions. From a biological perspective, earlier stages characterized by circulating tumor cell (CTC) dissemination, bone marrow seeding, and disseminated tumor cell (DTC) dormancy may represent potential windows for earlier risk assessment and more precise disease monitoring. However, the clinical exploitation of these windows remains largely theoretical, and prospective evidence is still required before CTC/DTC-informed early intervention can be incorporated into routine management. Consequently, shifting the timing of bone metastasis risk identification from “imaging-visible” to “biologically detectable” has emerged as a key objective in precision oncology.

Distant metastasis is a multistep, selection-driven cascade process. A central transition within this cascade occurs between circulating tumor cells (CTCs) and disseminated tumor cells (DTCs). CTCs are intact tumor cells shed from primary tumors or existing metastatic sites into the bloodstream and represent a detectable form of metastasis “in progress.” In a subset of patients, CTCs can be identified prior to clinical diagnosis, suggesting that dissemination may occur at early stages of disease development [7], [8]. Nevertheless, CTCs are exposed to multiple hostile stresses in the circulation—including hemodynamic shear forces, immune surveillance mediated by natural killer cells and T lymphocytes, and oxidative stress—resulting in an extremely low survival rate. As a consequence, distant metastasis is a biologically inefficient yet clinically consequential event [9].

To enhance survival and dissemination efficiency in the circulation, CTCs activate a range of adaptive programs. Through epithelial–mesenchymal transition (EMT), CTCs acquire increased migratory and invasive capacities, resistance to anoikis, and partial stem-like properties [10]. CTCs may form homotypic clusters or heterotypic aggregates with blood components such as platelets and neutrophils, thereby promoting survival through physical protection, immune evasion, and pro-survival signaling support [11], [12].

Despite these adaptive mechanisms, the vast majority of CTCs are eliminated during circulation. Only a small fraction of surviving cells successfully extravasate and persist in distant tissues. Among these, the bone marrow represents one of the most prominent colonization sites, where tumor cells are defined as disseminated tumor cells (DTCs) [13].

In contrast to CTCs, the biological properties of DTCs are highly dependent on their local microenvironment. Hallmark features of DTCs include long-term survival, reversible growth arrest, and reduced sensitivity to multiple therapeutic modalities [14]. Within specific niches, these properties enable DTCs to enter a dormant state that may persist for years or even decades, thereby constituting a critical cellular reservoir for late metastatic relapse [15]. When the bone marrow microenvironment is altered, or when DTCs acquire additional genetic or epigenetic changes that disrupt the existing balance, dormant cells may become reactivated and progress into clinically detectable metastatic lesions [16]. Thus, the transition from CTCs to DTCs is not merely a spatial relocation but a dynamic evolutionary process characterized by sequential phases of selection, adaptation, latency, and reactivation. This temporal progression underscores that bone metastasis should be interpreted as a time-resolved process rather than a static endpoint.

Bone metastasis–associated complications fundamentally arise from dysregulated bone remodeling driven by sustained interactions between tumor cells and the bone microenvironment. Previous studies have investigated the mechanisms of bone metastasis from multiple perspectives, including molecular signaling pathways, metabolic reprogramming, and the structural organization of the bone marrow niche [17]. However, the continuous cascade encompassing “CTC dissemination–bone-specific homing–DTC establishment–dormancy/reactivation state transitions” has not been systematically integrated within a unified CTC/DTC-centered framework. In particular, how CTCs acquire bone tropism and how bone marrow–derived signals influence the maintenance of DTC dormancy or their reactivation remain incompletely understood.

Based on this background, the present review proposes an integrative approach centered on CTCs and DTCs in bone metastasis. At the mechanistic level, we summarize the key regulatory nodes governing CTC dissemination, bone marrow colonization, DTC dormancy, and reactivation. From a translational perspective, we evaluate the clinical utility and limitations of CTC/DTC analyses in risk stratification, prognostic assessment, and treatment monitoring, and discuss emerging strategies, including single-cell multi-omics, spatial omics, and biomimetic bone microenvironment models.

## 2 A CTC/DTC-centered view of the bone-metastatic cascade

Bone metastasis is a multistep, selection-driven cascade shaped by the interaction between metastatic tumor cells and permissive organ microenvironments. In line with the classical “seed and soil” hypothesis, bone represents a preferred metastatic site because of its unique tissue architecture, active remodeling, and specialized marrow niches [18].

The bone marrow microenvironment is characterized by extensive vascularization, active remodeling, and tightly coupled interactions among osteoblasts, osteoclasts, stromal cells, endothelial cells, and immune components [19]. This dynamic niche not only supports CTC adhesion, extravasation, and early colonization, but also regulates DTC dormancy, therapy resistance, and reactivation, thereby determining whether metastasis remains microscopic or progresses to clinically overt disease. Importantly, bone metastasis is not necessarily a terminal stage. Tumor cells may re-enter the circulation from bone lesions, contributing to secondary dissemination and systemic relapse [20]. Accordingly, the bone marrow can be viewed as a regulatory hub coordinating dissemination and re-dissemination.

**Fig. 1:**
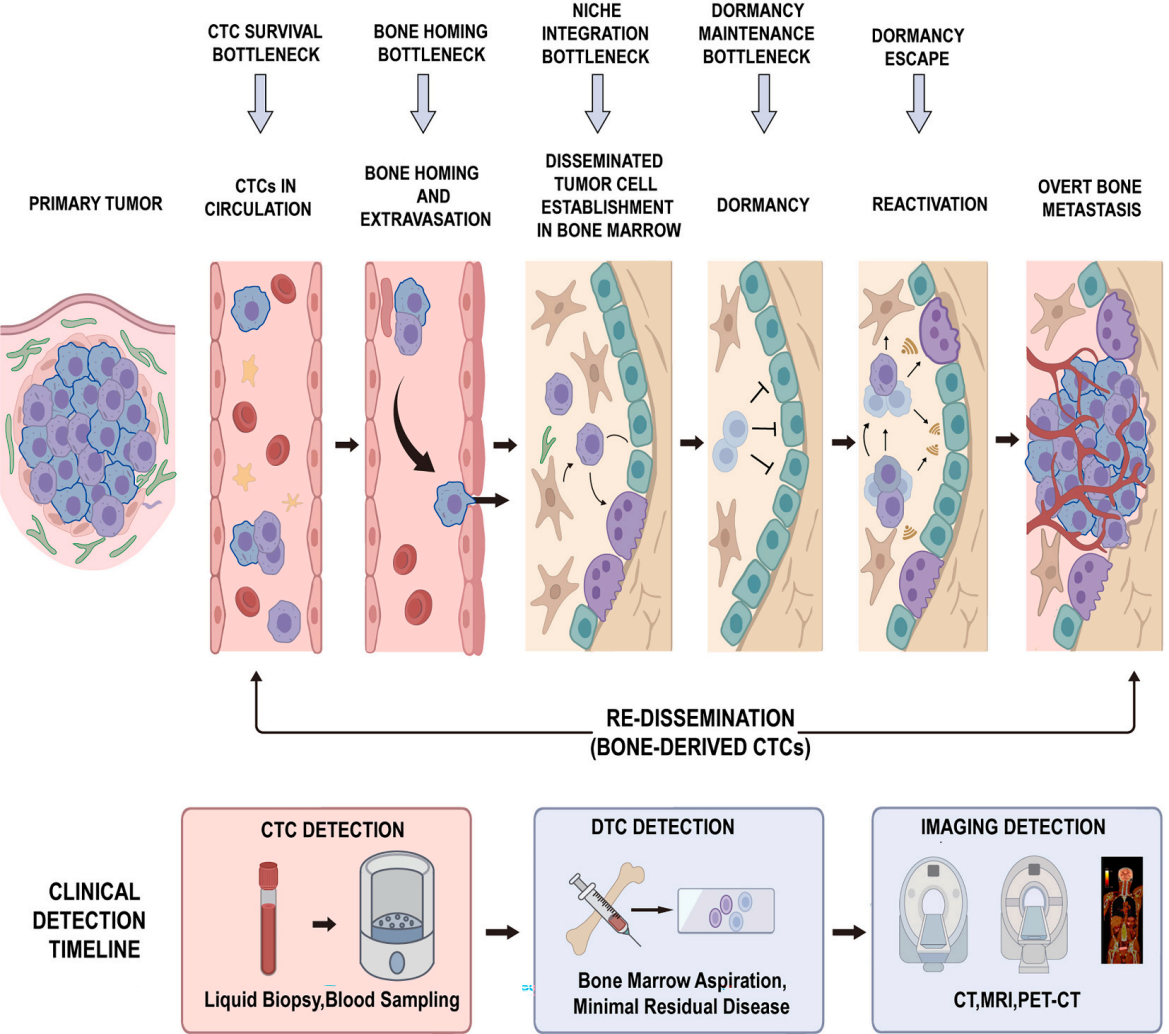
**Temporal cascade of bone metastasis and clinical detection windows of CTCs and DTCs.** Bone metastasis develops through a series of temporally ordered and selection-driven transitions rather than as a single deterministic event. Tumor cells shed from the primary tumor enter the circulation as circulating tumor cells (CTCs), where they encounter hemodynamic stress and immune surveillance. Only a small fraction survive this stage (CTC survival bottleneck) and subsequently undergo bone homing and extravasation into the bone marrow microvasculature (bone homing bottleneck). Following extravasation, tumor cells interact with stromal and vascular components of the bone marrow niche to establish disseminated tumor cells (DTCs), representing a second major selective checkpoint (niche integration bottleneck). Most established DTCs enter a long-term dormant state characterized by proliferative arrest and microenvironmental constraint (dormancy maintenance bottleneck). Under specific intrinsic or microenvironmental changes, a subset of dormant DTCs may escape dormancy and resume proliferation (dormancy escape), ultimately leading to overt bone metastatic lesions. Tumor cells within bone metastases may also re-enter the circulation as bone-derived CTCs, contributing to secondary dissemination and systemic relapse. The lower panel illustrates the relative clinical detection windows across disease stages: CTC detection through liquid biopsy reflects ongoing dissemination dynamics, DTC detection in bone marrow corresponds to minimal residual disease (MRD) and long-term relapse risk, whereas imaging modalities (CT, MRI, PET–CT) typically identify metastases only after macroscopic lesions have formed.

In this section, we conceptualize the metastatic cascade as a series of temporally ordered selection bottlenecks that shape tumor cell fate. At each transition—from circulation to bone homing, from extravasation to establishment, and from dormancy to reactivation—most cells are eliminated, whereas a small subset adapts to increasingly restrictive microenvironmental constraints. This time-resolved framework provides the conceptual basis for the following sections and is schematically summarized in Fig. 1.

### 2.1 Bone homing of circulating tumor cells

Circulating tumor cells (CTCs) do not disseminate randomly within the bloodstream but instead exhibit organ tropism, with bone representing a preferred homing site. Bone homing is often preceded by remote remodeling of the bone marrow microenvironment, driven by tumor-derived factors such as granulocyte colony-stimulating factor (G-CSF), vascular endothelial growth factor A (VEGF-A), and transforming growth factor-β, which collectively contribute to pre-metastatic niche formation [21].

At the mechanistic level, CTC homing to bone involves coordinated sensing of chemokine gradients, adhesion cues, and endothelial signals, partially resembling hematopoietic stem cell homing [22]. Under physiological shear stress, CTCs undergo sequential processes of endothelial capture, transient interaction, and integrin-mediated stabilization, ultimately enabling transendothelial migration into the bone marrow parenchyma. After extravasation, CTCs enter the bone marrow compartment and are defined as disseminated tumor cells (DTCs) [23]. Notably, only a small fraction of CTCs successfully complete this transition, highlighting bone homing as an early rate-limiting step in the metastatic cascade (Fig. 1).

#### 2.1.1 Genetic and epigenetic regulation

The bone-tropic homing capacity of circulating tumor cells (CTCs) is not acquired randomly but is shaped by the combined influence of their genetic and epigenetic backgrounds. Structural alterations in specific chromosomal regions are significantly associated with an increased propensity for bone metastasis. For example, amplification of the 16q23 locus and activation of the associated transcriptional programs in breast cancer have been shown to enhance tumor cell responsiveness to bone marrow–derived chemotactic cues and adhesion molecules [24].

At the gene level, aberrant expression of molecules such as TFF1, ITGA5, and ZNF217 has been associated with bone metastasis. These genes are predominantly involved in cell adhesion, interactions with the bone matrix, and migration-related processes [25], [26], [27]. Transcriptomic analyses have identified a bone-tropic gene signature comprising MAF, CAPG, GIPC1, and IL1B, supporting a molecular basis for CTC adaptation to the bone microenvironment [28].

Despite strong associations between multiple genes and bone metastasis risk, no single “bone metastasis–specific” driver mutation has been identified to date, suggesting that bone homing reflects a coordinated, multi-gene adaptive process rather than a solitary driving event [29]. In this context, epigenetic regulation is increasingly recognized as a critical layer linking genetic background to microenvironmental responsiveness. Epigenetic alterations, including DNA methylation and histone modifications, dynamically regulate genes involved in chemotaxis, adhesion, and cell fate determination. These changes confer enhanced phenotypic plasticity upon CTCs without altering the underlying DNA sequence [30], [31]. In clear cell renal cell carcinoma, epigenetic reprogramming amplifies VHL–HIF signaling and upregulates receptors such as CXCR4, enhancing tumor cell homing to the bone marrow [32].

Collectively, these findings indicate that bone tropism of CTCs is not stochastic but is partially “pre-programmed” at the primary tumor stage and subsequently refined under selective pressures in circulation and within the bone marrow microenvironment. This “pre-programming followed by selection” model provides a conceptual framework for understanding organ-specific metastasis and its partial predictability. This model suggests that bone tropism emerges through iterative selection across temporal stages rather than being determined at a single event.

#### 2.1.2 Key receptors and signaling pathways

The ability of circulating tumor cells (CTCs) to sense and respond to bone marrow–derived cues is mediated by cell surface receptors and their downstream signaling pathways, among which chemokine axes play a central role in bone homing. The CXCR4–CXCL12 axis is one of the most extensively studied chemotactic pathways in this context. Its ligand CXCL12 is highly expressed in the bone marrow and forms a stable gradient that guides CTCs toward vascular regions under shear stress conditions [33]. CXCL12 can act synergistically with endothelial P-selectin to enhance initial CTC capture and transient retention within bone marrow sinusoids, thereby facilitating extravasation [34]. In breast cancer, CX3CR1-expressing CTCs enhance retention in bone tissue through interaction with endothelial fractalkine, suggesting that distinct chemokine axes exert complementary roles during bone homing [35].

While chemotactic signaling provides directional guidance, stable arrest and extravasation within bone marrow sinusoids depend primarily on adhesion molecule–mediated interactions with the endothelium and extracellular matrix, together with downstream signal integration [33], [34], [36]. Integrins such as αvβ3 and α4β1 enable CTCs to engage bone marrow ligands including osteopontin, fibronectin, and VCAM-1, permitting firm adhesion under shear stress and enhancing bone homing efficiency [36]. Thus, chemokine signaling provides directional cues, whereas adhesion molecules and microenvironmental signals are required for stable arrest and effective colonization.

In addition to classical chemokine axes, the RANKL/RANK pathway may also function as a chemokine-like migration signal in selected cancers. Although RANKL is traditionally recognized as a key osteoclastogenic factor in bone remodeling, it can directly promote the migration of RANK-expressing tumor cells and contribute to tissue-specific metastatic behavior toward bone. This mechanism suggests that bone-derived differentiation factors may not only regulate osteoclast activity but also provide directional and pro-migratory cues for tumor cells during bone homing [37].

**Fig. 2:**
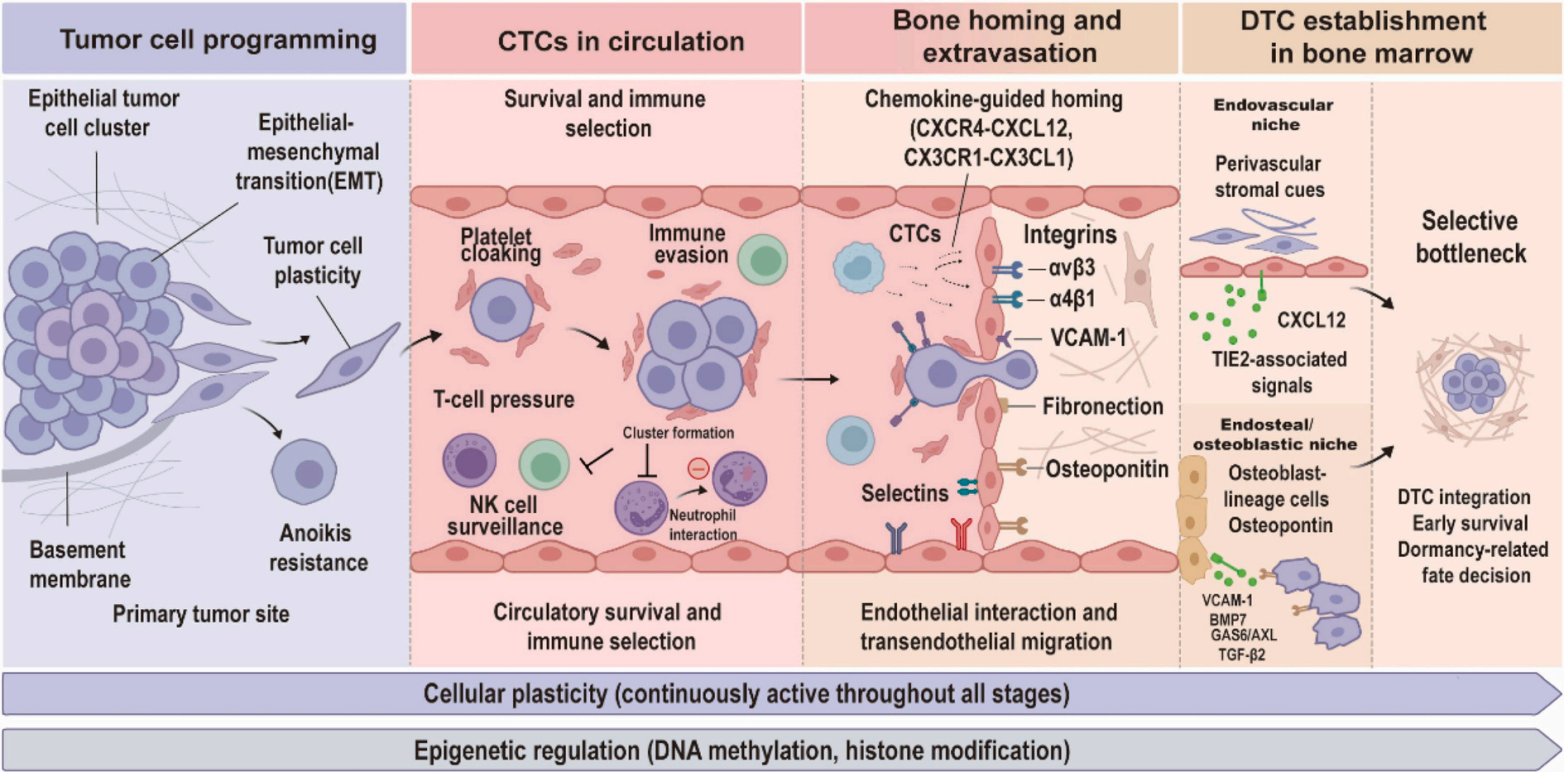
**Molecular, immune, and niche-regulated mechanisms underlying the CTC-to-DTC transition during bone metastasis.** The transition from circulating tumor cells (CTCs) to disseminated tumor cells (DTCs) is governed by coordinated molecular, immune, and niche-dependent mechanisms that enable tumor cell detachment, survival in circulation, bone homing, extravasation, and establishment within the bone marrow. At the primary tumor site, epithelial–mesenchymal transition (EMT), tumor cell plasticity, anoikis resistance, and intravasation facilitate the generation of CTCs. In the circulation, CTCs encounter substantial immune and mechanical selective pressures. Platelet cloaking, neutrophil interaction, CTC cluster formation, and immune evasion may enhance CTC survival, whereas natural killer cell surveillance and cytotoxic T-cell pressure contribute to CTC elimination. Bone homing and extravasation are mediated by chemokine-guided migration and endothelial adhesion, including CXCR4–CXCL12, CX3CR1–CX3CL1, selectins, integrins such as αvβ3 and α4β1, VCAM-1, fibronectin, and osteopontin. Following transendothelial migration, DTCs may localize to spatially distinct bone marrow niches, including endovascular niches and endosteal/osteoblastic niches. These niches provide endothelial, perivascular, osteoblastic, adhesive, and dormancy-associated cues that support DTC niche integration, early survival, and dormancy-related fate decisions. Cellular plasticity and epigenetic regulation remain active across all stages of this transition.

Beyond chemotactic and adhesive signaling, growth factors and inflammation-related pathways further amplify CTC bone tropism through cross-regulatory mechanisms. Activation of platelet-derived growth factor receptor α (PDGFRα) enhances bone homing of prostate cancer CTCs and strengthens microenvironmental interactions by upregulating inflammatory mediators such as IL-1β [38]. In animal models, blockade of IL-1R signaling reduces bone metastasis incidence, supporting an amplifying role of inflammation-related pathways in bone homing and early colonization [39]. Together, these signaling modules coordinate directional migration, stable adhesion, and microenvironmental amplification, thereby shaping the efficiency of CTC bone homing (Fig. 2).

### 2.2 Establishment of disseminated tumor cells

Once circulating tumor cells (CTCs) traverse the vascular barrier and enter the bone marrow parenchyma, they are defined as disseminated tumor cells (DTCs). However, entry into the bone marrow does not equate to successful metastatic establishment. This stage represents a major selective bottleneck, as the majority of DTCs are eliminated by mechanical stress, immune clearance, or insufficient niche support, whereas only a small subset acquires long-term survival capacity [40].

Initial DTC establishment relies on stable interactions with the bone marrow extracellular matrix and stromal cells. Integrin-mediated interactions play a central role in this process. For example, integrin αvβ3 mediates binding to osteopontin and fibronectin, whereas α4β1 promotes anchorage through interactions with VCAM-1 and ICAM-1 [36]. Additional molecules, including Annexin II and E-cadherin, further contribute to DTC retention within bone marrow niches [41], [42].

Importantly, establishment extends beyond adhesion and involves a second layer of selection within the bone marrow niche. Successfully retained DTCs must acquire survival advantages, including metabolic adaptation, immune evasion, and integration of signals from osteoblasts, endothelial cells, and immune components. Clinical observations show that DTC detection rates in bone marrow exceed the incidence of overt metastasis, indicating that while the niche permits survival, it simultaneously constrains proliferative outgrowth and governs subsequent fate decisions toward dormancy or reactivation [43], [44].

DTC establishment therefore represents a transition from physical arrival to niche integration and functions as a critical checkpoint that determines whether disseminated cells enter long-term dormancy or progress toward metastatic outgrowth (Fig. 1). As summarized in Fig. 2, the CTC-to-DTC transition involves not only vascular arrest and extravasation, but also immune-mediated selection and subsequent localization within endovascular or endosteal/osteoblastic niches. These distinct microenvironmental contexts shape early DTC survival, niche integration, and dormancy-related fate decisions.

### 2.3 Dormancy of disseminated tumor cells

Following establishment within the bone marrow, disseminated tumor cells (DTCs) predominantly adopt a long-term, reversible state characterized by low or absent proliferation, commonly referred to as dormancy. Rather than a passive condition, dormancy represents an actively maintained state governed by coordinated cell-intrinsic programs and microenvironmental signals. This state is defined by suppressed proliferation, sustained survival capacity, and relative insensitivity to multiple therapeutic modalities [45], [46]. In this context, dormancy can be conceptualized as a regulated equilibrium rather than a transient interruption of proliferation.

At the cell-intrinsic level, dormancy is maintained by regulatory programs that enforce cell-cycle restriction and phenotypic stability. For example, FBXW7 limits proliferative signaling through degradation of pro-growth proteins, whereas the transcription factor NR2F1 orchestrates dormancy-associated transcriptional programs that stabilize quiescence; disruption of these pathways may facilitate exit from dormancy and promote metastatic progression [46], [47].

**Fig. 3:**
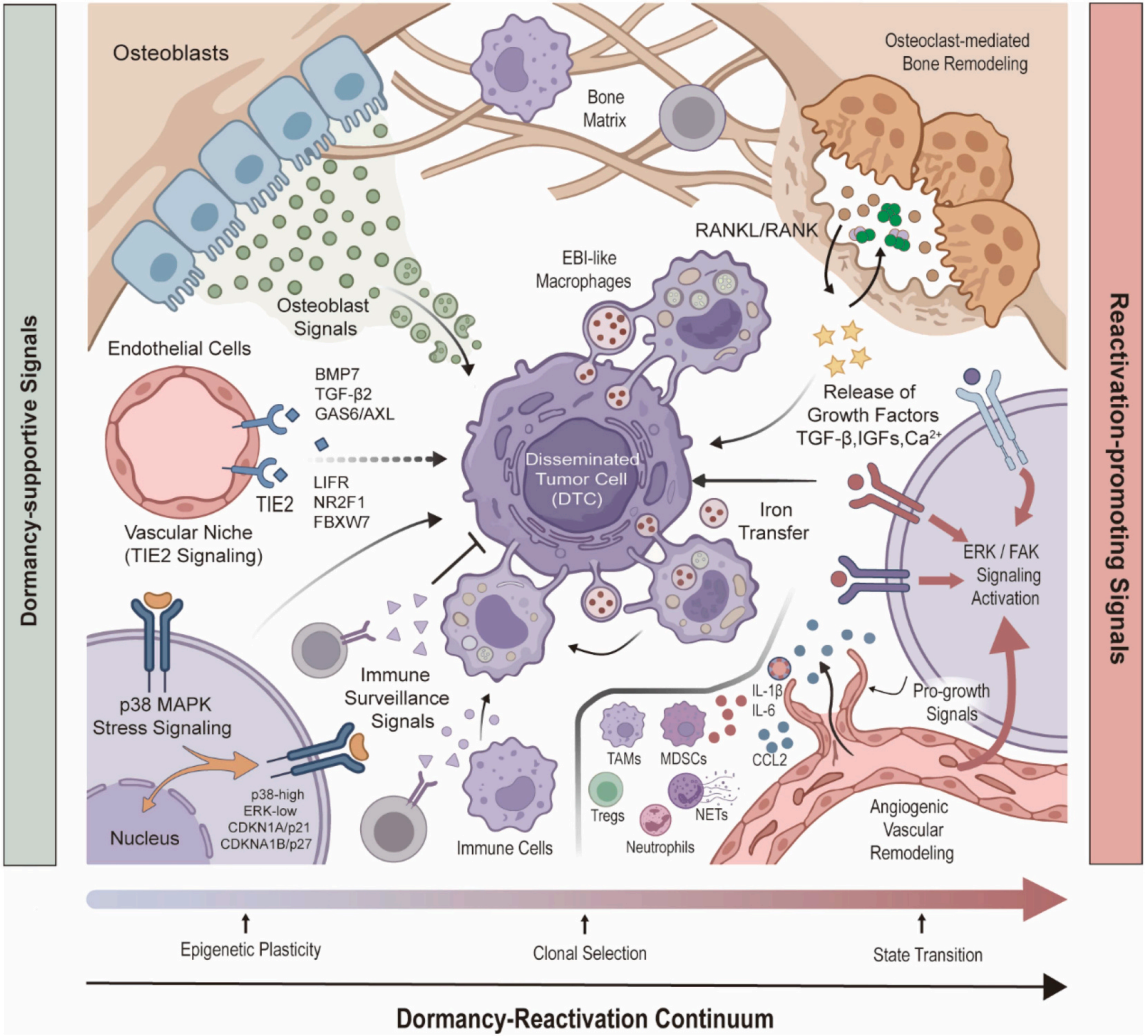
**Molecular and immunological regulation of DTC dormancy and reactivation in the bone marrow niche.** The fate of disseminated tumor cells (DTCs) within the bone marrow is regulated by a dynamic balance between dormancy-supportive signals and reactivation-promoting signals. Dormancy-supportive cues arise from osteoblast-lineage cells, vascular niches, immune surveillance mechanisms, and stress-adaptive signaling pathways. Osteoblast-derived factors such as BMP7, TGF-β2, and GAS6/AXL-related signals, together with LIFR, NR2F1, FBXW7, TIE2 signaling, p38 MAPK activation, ERK suppression, and cell-cycle inhibitory programs involving CDKN1A/p21 and CDKN1B/p27, contribute to DTC quiescence and long-term persistence. Immune surveillance may further restrain proliferative outgrowth and maintain disseminated cells in a dormant or equilibrium-like state. In contrast, reactivation-promoting signals are associated with osteoclast-mediated bone remodeling, RANKL/RANK signaling, release of bone matrix-derived factors such as TGF-β, IGFs, and Ca^2+^, angiogenic vascular remodeling, inflammatory cytokines, and immune-suppressive cell populations. Tumor-associated macrophages, myeloid-derived suppressor cells, regulatory T cells, neutrophils, and neutrophil extracellular traps may contribute to a permissive microenvironment for DTC survival and reactivation. EBI-like macrophages may provide metabolic support through iron transfer, facilitating adaptation to the hypoxic bone marrow niche. These molecular, immunological, metabolic, and vascular cues collectively drive the dormancy–reactivation continuum, in which epigenetic plasticity, clonal selection, and state transitions determine whether DTCs remain clinically silent or progress toward overt bone metastasis.

Microenvironmental signals further reinforce dormancy. Bone marrow vascular–associated TIE2 signaling induces the expression of cyclin-dependent kinase inhibitors CDKN1A and CDKN1B, leading to G0/G1 arrest and sustained survival within the niche [48]. Dormancy maintenance is also shaped by a balance between niche-derived suppressive cues and immune-mediated equilibrium. Osteoblast- and stromal-derived factors such as TGF-β2, BMP7, GAS6/AXL, and LIFR-associated signaling can activate stress-adaptive pathways, including p38 MAPK, and promote the expression of cell-cycle inhibitory programs, thereby reinforcing quiescence and limiting proliferative expansion [49], [50], [51], [52], [53], [54]. In parallel, cytotoxic immune surveillance mediated by natural killer cells and T lymphocytes may eliminate highly proliferative or immunogenic disseminated cells, leaving behind slow-cycling or immune-evasive DTC subpopulations that are more compatible with long-term persistence [40], [44]. Thus, DTC dormancy reflects not only tumor-intrinsic cell-cycle restraint, but also an equilibrium imposed by osteoblastic, vascular, stromal, and immune components of the bone marrow niche. These observations highlight that dormancy is not solely governed by tumor-intrinsic programs but is tightly regulated by niche-derived signals within the bone marrow microenvironment (Fig. 3).

Dormancy does not necessarily entail loss of differentiation markers. In breast cancer models, dormant DTCs can retain epithelial-associated markers such as HER2 and CK19 in the absence of proliferative activity, indicating that long-term latency can coexist with partial lineage identity [55].

Functionally, dormancy represents a trade-off state: while suppression of proliferation limits immediate metastatic burden, it simultaneously enables immune evasion and resistance to therapy, thereby preserving the potential for future outgrowth.

Importantly, dormancy may represent the default fate of the majority of DTCs that successfully establish within the bone marrow niche. From this perspective, metastatic outgrowth is better understood not as an inevitable consequence of dissemination, but as a context-dependent escape from dormancy constraints. Accordingly, bone metastasis progression can be conceptualized as a dynamic balance between dormancy maintenance and proliferative escape, rather than continuous tumor expansion.

### 2.4 Reactivation of disseminated tumor cells and metastatic progression

Although disseminated tumor cells (DTCs) can maintain long-term dormancy within the bone marrow, this state is not irreversible. Reactivation should not be interpreted simply as the resumption of proliferation, but rather as an escape from dormancy-maintaining constraints imposed by both cell-intrinsic programs and the surrounding microenvironment.

From a microenvironmental perspective, reactivation is frequently associated with disruption of dormancy-supportive niche conditions. During bone remodeling, shifts in the balance between osteoblast and osteoclast activity alter extracellular matrix composition, mechanical properties, and soluble factor availability, thereby weakening suppressive signals and releasing pro-proliferative cues [49]. In parallel, vascular remodeling and increased angiogenic signaling may convert previously restrictive vascular niches into more permissive environments that favor DTC outgrowth [56]. Immune and inflammatory remodeling further contributes to this permissive switch. Tumor-associated macrophages, myeloid-derived suppressor cells, regulatory T cells, and neutrophils can weaken immune surveillance and create a pro-inflammatory microenvironment that favors DTC survival and expansion [57], [58], [59], [60]. Cytokine and chemokine pathways, including IL-1β/IL-1R, IL-6/STAT3, CCL2/CCR2, CXCL12/CXCR4, and CX3CL1/CX3CR1, may promote tumor cell survival, stromal recruitment, osteoclast activation, and bone-directed migration [33], [35], [38], [39], [57], [58], [59]. In addition, neutrophil extracellular traps can remodel the extracellular matrix and have been implicated in awakening dormant cancer cells under inflammatory conditions [61]. These inflammatory and immune-suppressive signals cooperate with osteoclast-mediated bone resorption and angiogenic remodeling to facilitate the transition from dormancy maintenance to metastatic outgrowth.

At the intracellular level, the transition from dormancy to reactivation is governed by shifts in signaling balance rather than activation of a single pathway. In particular, the balance between p38 MAPK and ERK/FAK-associated signaling has been proposed as an important regulatory node in the dormancy–reactivation transition. Elevated p38 activity is generally associated with stress adaptation and proliferative restraint, whereas increased ERK/FAK-related signaling is linked to adhesive, mechanotransductive, and pro-growth programs that may favor exit from quiescence and metastatic outgrowth [50], [51].

Importantly, reactivation is not a synchronized event across the dormant DTC population but instead reflects a process of clonal selection under changing environmental conditions. Single-cell analyses indicate that only a subset of DTCs possesses the capacity to undergo state transitions in response to specific microenvironmental cues [62]. This competence is likely determined by pre-existing epigenetic states, metabolic adaptability, and patterns of niche interaction, suggesting that metastatic outgrowth arises from selective expansion of permissive clones rather than uniform activation of all dormant cells (Fig. 3).

Conceptually, DTC reactivation represents a critical transition from a stable dormancy equilibrium to proliferative escape. This shift underlies the progression from clinically occult disease to overt metastasis and provides a mechanistic explanation for late relapse occurring years after primary treatment. Accordingly, the dormancy–reactivation interface represents a temporally restricted yet biologically pivotal window for mechanistic investigation, risk monitoring, and potential therapeutic intervention. Rather than a deterministic outcome, metastatic progression may therefore be viewed as a probabilistic event governed by the dynamic balance between dormancy maintenance and escape.

### 2.5 Bone microenvironmental interactions and the vicious cycle

Progression of bone metastasis is not determined solely by tumor cell–intrinsic properties but instead reflects dynamic, bidirectional interactions between tumor cells and the bone microenvironment. The classical “vicious cycle” model describes a self-reinforcing loop in which tumor cells stimulate osteoclast activity, leading to bone resorption and the release of growth factors that further promote tumor expansion [63]. However, this framework primarily captures late-stage osteolytic progression. Increasing evidence indicates that the bone microenvironment functions as an active regulator across multiple stages of the metastatic cascade, shaping tumor cell survival, dormancy maintenance, and reactivation (Fig. 3).

At early stages of bone metastasis, signaling interactions between tumor cells and bone-remodeling cells, including osteoclasts and osteoblasts, are already altered. These changes are closely linked to imbalances in osteoblast–osteoclast coupling, which influence both the intensity of bone remodeling and the production of local niche-derived signals. Tumor cell–secreted osteoclast-activating factors enhance osteoclast differentiation and activity, resulting in degradation of the bone matrix and release of growth factors such as transforming growth factor-β (TGF-β), which subsequently promote tumor cell proliferation and migration [64]. Concurrently, suppression or dysregulation of osteoblast function remodels the bone marrow niche and alters the survival and proliferative cues received by DTCs, thereby weakening dormancy-maintaining constraints and facilitating transition toward reactivation [57], [65], [66].

Beyond bone remodeling–associated cells, immune components of the bone microenvironment also contribute to this regulatory network. Tumor-associated macrophages and other immune cells can release inflammatory mediators that attenuate dormancy-supportive signals while enhancing pro-growth and pro-migratory pathways, thereby amplifying bone metastatic progression [58], [59].

Recent studies further suggest that immune components regulate bone metastasis not only through inflammatory signaling but also through metabolic coupling between tumor cells and the bone marrow niche. A specialized population of bone marrow macrophages resembling erythroblastic island (EBI) macrophages has been shown to accumulate within metastatic niches, where they function as iron donors to tumor cells. Through iron transfer, these macrophages support tumor cell survival and metabolic adaptation to the hypoxic bone microenvironment while simultaneously disrupting normal erythropoiesis. This finding highlights a previously underappreciated metabolic coupling between tumor cells and the bone marrow niche, suggesting that nutrient availability, in addition to signaling interactions, can critically influence metastatic progression [60].

Importantly, the influence of the bone microenvironment is not confined to local lesions. Tumor cells exposed to bone-derived signals may undergo phenotypic reprogramming, acquire enhanced invasive capacity, and re-enter the circulation as circulating tumor cells (CTCs) with increased metastatic potential, thereby contributing to secondary dissemination and systemic disease progression [20].

Collectively, bone microenvironmental interactions should be viewed not as a simple linear “vicious cycle,” but as a dynamic regulatory network that governs DTC dormancy, reactivation, and metastatic outgrowth. By modulating the balance between dormancy maintenance and proliferative escape, the bone microenvironment represents both a driver of disease evolution and a potential source of stage-specific therapeutic vulnerabilities.

### 2.6 Cellular and molecular regulation of the bone metastatic niche

The bone metastatic niche is composed of multiple spatially and functionally distinct microenvironments, including endosteal/osteoblastic niches, vascular niches, osteoclast-enriched remodeling niches, and immune-regulatory niches [17], [44], [49], [56], [64]. These compartments do not act independently; rather, they generate overlapping cytokine, chemokine, adhesive, and metabolic signals that collectively determine whether newly established DTCs undergo immune clearance, long-term dormancy, or metastatic outgrowth [44], [64].

Within the endosteal and osteoblastic niche, osteoblast-lineage cells and bone marrow stromal cells provide both adhesive and soluble cues that influence DTC fate [49], [65]. Adhesion molecules such as osteopontin, VCAM-1, and fibronectin facilitate DTC anchorage [36], whereas osteoblast-derived dormancy-associated signals, including GAS6/AXL, BMP7, TGF-β2, and LIFR-related pathways, may promote cell-cycle restraint and stress adaptation [51], [52], [53], [54]. These signals converge on downstream programs such as p38 MAPK activation, ERK suppression, and increased expression of cyclin-dependent kinase inhibitors, thereby supporting a quiescent or slow-cycling state [48], [50], [51], [52], [54]. In this context, the osteoblastic niche functions not only as a physical docking site but also as an active regulatory compartment that can impose dormancy on disseminated tumor cells [49], [65].

The vascular niche provides a second regulatory layer. Bone marrow endothelial cells, perivascular stromal cells, and sinusoidal vessels produce chemokines and adhesion signals, including CXCL12, selectins, VCAM-1, and TIE2-associated cues, which regulate CTC arrest, extravasation, and early DTC survival [33], [34], [48], [56]. Stable or quiescent vascular niches may reinforce dormancy, whereas angiogenic remodeling can convert the local microenvironment into a more permissive state for metastatic outgrowth [48], [56]. This vascular switch is particularly relevant because DTCs often reside in close proximity to bone marrow microvessels, where subtle changes in endothelial activation, permeability, and perivascular cytokine production may alter the balance between dormancy maintenance and reactivation [44], [56].

In contrast, osteoclast-enriched remodeling niches are closely linked to dormancy escape and overt metastatic progression [63], [64]. Tumor-derived factors stimulate osteoclast differentiation and bone resorption through pathways such as RANKL/RANK, IL-6, IL-11, PTHrP, and TGF-β-related signaling [37], [63], [64]. Bone matrix degradation releases stored growth factors, including TGF-β and IGFs, and increases local calcium availability, thereby generating pro-survival and pro-proliferative signals for tumor cells [63], [64]. These mechanisms amplify the classical vicious cycle and provide a molecular explanation for how changes in bone remodeling can shift DTCs from a constrained dormant state toward proliferative expansion [58], [63], [64].

Immune components further shape the niche-dependent fate of DTCs [40], [57], [58], [59]. Natural killer cells and cytotoxic T lymphocytes may eliminate disseminated cells or maintain them in an equilibrium state [40], whereas tumor-associated macrophages, myeloid-derived suppressor cells, regulatory T cells, and neutrophils can create an immunosuppressive and pro-inflammatory environment that favors DTC persistence and reactivation [57], [58], [59], [60]. Cytokine and chemokine axes such as IL-1β/IL-1R, IL-6/STAT3, CCL2/CCR2, CXCL12/CXCR4, and CX3CL1/CX3CR1 can promote tumor cell survival, immune evasion, stromal recruitment, and bone-directed migration [33], [35], [38], [39], [57], [58], [59]. In addition, neutrophil extracellular traps and macrophage-mediated metabolic support may remodel extracellular matrix architecture or nutrient availability, thereby facilitating the transition from dormancy to outgrowth [60], [61].

Taken together, niche-driven dormancy and metastatic reactivation are regulated by coordinated interactions among osteoblastic, vascular, osteoclastic, stromal, and immune compartments [44], [49], [56], [64]. Rather than acting as a single permissive “soil,” the bone marrow niche should be viewed as a dynamic regulatory ecosystem in which cytokine/chemokine gradients, adhesion programs, immune pressure, and bone remodeling collectively shape CTC-to-DTC transition, dormancy maintenance, and metastatic escape.

## 3 Clinical detection and translational value of CTCs and DTCs

The clinical relevance of circulating tumor cells (CTCs) and disseminated tumor cells (DTCs) lies in providing time-resolved biological information that complements imaging-based diagnosis. By reflecting dissemination dynamics, dormant residual disease, and potential reactivation before radiographic manifestation, CTCs and DTCs may refine metastatic risk assessment and treatment monitoring.

In clinical settings, CTCs and DTCs represent distinct temporal dimensions of the metastatic process. CTCs reflect active systemic dissemination, and their abundance, phenotypic characteristics, and molecular features correlate with disease activity and metastatic potential [67]. In contrast, DTCs residing in the bone marrow correspond more closely to the cellular substrate of minimal residual disease (MRD) and serve as indicators of long-term relapse risk [68]. This temporal complementarity underlies their potential synergistic value in bone metastasis management. Key attributes of CTCs and DTCs across disease stages are summarized in Table 1.

**Table 1:** Clinical interpretation of CTCs and DTCs across temporal stages of bone metastasis.

Temporal stage	Cellular readout	Biological meaning	Clinical utility	Key limitations
Systemic dissemination	CTC enumeration and dynamics	Real-time dissemination activity	Risk stratification; prognosis	High heterogeneity; limited bone specificity
Bone homing and colonization	Bone-tropic CTC phenotypes	Increased propensity for bone seeding	Early warning of bone metastasis risk	Lack of standardized definitions
Dormancy / MRD	Bone marrow DTC persistence	Long-term survival in niche	Relapse risk assessment	Invasive sampling; low feasibility
Reactivation	CTC/DTC molecular shifts	Exit from dormancy	Monitoring resistance risk	Primarily correlative evidence

**Abbreviations:** CTC, circulating tumor cell; DTC, disseminated tumor cell; MRD, minimal residual disease.

**Note:** CTCs primarily reflect dissemination dynamics and provide short-term prognostic information, whereas DTCs correspond to minimal residual disease and long-term relapse risk. Current evidence supports their complementary use rather than replacement of imaging or direct therapeutic decision-making.

Extensive clinical evidence demonstrates that CTC detection rates and counts are associated with unfavorable prognosis across multiple solid tumor types. However, CTC enumeration alone cannot fully capture the biological complexity of bone metastasis and is limited by substantial heterogeneity and dynamic fluctuation. By comparison, although DTC detection is constrained by invasiveness and procedural complexity, it provides unique insight into long-term latency and relapse risk. Rational integration of CTC- and DTC-derived information therefore represents a central challenge in translating these biomarkers into clinically actionable strategies.

Accordingly, this section reviews current evidence regarding CTC/DTC applications in bone metastasis risk stratification, prognostic assessment, and treatment monitoring, and discusses their positioning within future precision management frameworks.

### 3.1 Risk prediction and early warning of bone metastasis

Bone metastasis typically develops through prolonged latency and gradual evolution; however, current clinical practice relies predominantly on imaging modalities that detect already established lesions, limiting timely assessment of underlying biological risk. In this context, detection of circulating tumor cells (CTCs) and disseminated tumor cells (DTCs) provides complementary biological information, enabling risk stratification and identification of high-risk populations prior to radiographic manifestation [69], [70].

At the systemic level, multiple studies have demonstrated that CTC detection rates and counts are associated with metastatic risk and unfavorable prognosis across various solid tumor types, with bone metastasis representing a major clinical outcome [71], [72], [73]. Compared with single time-point measurements, longitudinal changes in CTC levels are more informative: persistent detection or increasing counts reflect heightened dissemination activity and are associated with increased risk of subsequent bone or multi-organ metastasis [74]. However, CTC-based prediction is influenced by detection platforms, tumor types, and disease stages, leading to variability across studies and limiting its robustness as a standalone clinical indicator [75], [76].

Another important limitation is the temporal uncertainty of CTC detection. CTC dissemination may occur very early during cancer evolution, even before radiographically detectable metastasis, but the release of CTCs into the bloodstream is often intermittent and their abundance is extremely low. Consequently, a single negative CTC test does not exclude ongoing dissemination or future metastatic risk, particularly in patients with occult or dormant disease [7], [8], [75], [76].

Beyond enumeration, molecular and phenotypic characterization of CTCs has demonstrated enhanced predictive potential. In this context, candidate markers related to epithelial identity, EMT/plasticity, stemness, bone homing, dormancy, and reactivation may provide more biologically informative readouts than CTC/DTC enumeration alone. Representative CTC/DTC markers with potential relevance to bone metastasis risk stratification and relapse prediction are summarized in Table 2. Subpopulations expressing chemokine receptors, adhesion molecules, or stemness-associated markers are more likely to participate in bone homing and establishment, thereby correlating with increased risk of bone metastasis [77]. These “functional CTC” features may more directly reflect metastasis-related biological behavior, although current evidence remains largely retrospective and lacks standardization.

**Table 2:** Candidate CTC/DTC markers with potential relevance to bone metastasis risk stratification and relapse prediction.

Marker category	Representative markers	Biological implication	Potential clinical relevance	Current limitations
Epithelial/tumor-identification markers	EpCAM, CK8/18/19, HER2	Identification of epithelial-derived tumor cells; partial maintenance of epithelial lineage identity	CTC/DTC detection and enrichment; assessment of tumor-cell origin	May miss EMT-like or EpCAM-low CTCs/DTCs; not bone-specific [67], [68], [76]
EMT and phenotypic plasticity markers	Vimentin, N-cadherin, TWIST, SNAIL, ZEB1	Increased motility, invasiveness, anoikis resistance, and therapy tolerance	Identification of more invasive or treatment-resistant CTC/DTC subpopulations	Marker expression is dynamic and platform-dependent; clinical thresholds remain undefined [9], [10], [85]
Stemness-associated markers	CD44^high^/CD24^low^, ALDH1, SOX2, NANOG	Self-renewal capacity, survival under stress, and metastatic competence	May help identify CTC/DTC subsets with long-term persistence or relapse potential	Limited specificity; functional validation in bone metastasis remains incomplete [9], [85]
Bone-homing and adhesion markers	CXCR4, CX3CR1, ITGA5, αvβ3, α4β1, VCAM-1-related interactions, RANK	Chemokine-guided migration, endothelial arrest, extracellular matrix adhesion, and bone marrow niche localization	Potential enrichment of CTCs/DTCs with bone-seeding propensity	Most evidence is tumor-type-specific or preclinical; clinical standardization is lacking [25], [28], [33], [34], [35], [36], [37]
Dormancy-associated markers	NR2F1, FBXW7, p38 MAPK activation, CDKN1A/p21, CDKN1B/p27, TIE2, GAS6/AXL, BMP7-related signaling, TGF-β2-related signaling	Cell-cycle restraint, stress adaptation, quiescence, and long-term niche persistence	May support identification of dormant DTCs and long-term relapse risk	Bone marrow sampling is invasive; dormancy markers are context-dependent and not yet validated for routine decision-making [46], [47], [48], [50], [51], [52], [53], [54]
Reactivation/outgrowth-associated markers	Ki-67, ERK/FAK activation, mTOR activation, IL-1β/IL-1R, IL-6/STAT3, MAF/CAPG/GIPC1/IL1B signature	Proliferative escape, inflammatory activation, bone-tropic fitness, and metastatic outgrowth	May indicate transition from dormant MRD toward active progression	Mostly correlative evidence; temporal validation in prospective cohorts is still needed [28], [38], [39], [50], [58], [59], [62]
Therapy-resistance markers	AR-V7, HER2 alterations, Akt pathway activation, therapy-relevant target shifts	Treatment adaptation and resistant tumor-cell states	Treatment monitoring and early detection of emerging resistance	Marker utility is tumor-specific and treatment-specific; not necessarily bone-metastasis-specific [55], [80], [81]

**Abbreviations:** CTC, circulating tumor cell; DTC, disseminated tumor cell; EMT, epithelial–mesenchymal transition; MRD, minimal residual disease.

**Note:** These markers should be interpreted as candidate biological indicators rather than established standalone clinical decision tools. Their predictive value for bone metastasis relapse requires prospective validation, standardized detection platforms, and integration with imaging and clinical risk factors.

In contrast, detection of DTCs in the bone marrow provides a more stable indicator of long-term relapse risk. Clinical studies have shown that DTCs can be detected even in radiographically negative stages or after apparent treatment response, and their persistence is significantly associated with subsequent bone metastasis and disease recurrence [78], [79]. These findings support the concept that DTCs represent a cellular correlate of minimal residual disease (MRD), indicating that metastatic risk may be biologically established well before clinical manifestation.

Nevertheless, the invasive nature and procedural complexity of DTC detection limit its routine clinical use, confining its application primarily to high-risk stratification or research settings [67]. By comparison, CTC detection enables repeatable sampling and dynamic monitoring but shows limited stability in predicting long-term outcomes. From a temporal perspective, CTCs primarily reflect ongoing dissemination activity, whereas DTCs capture the latent reservoir of relapse.

Overall, CTC/DTC analyses are unlikely to replace imaging-based assessment but may complement it by identifying biologically high-risk yet radiographically occult disease. Integration of CTC dynamics, functional phenotypes, and DTC status may enable development of refined, time-resolved risk stratification models and facilitate earlier, biology-informed intervention strategies.

### 3.2 Treatment monitoring and relapse prediction

Beyond risk prediction, circulating tumor cells (CTCs) and disseminated tumor cells (DTCs) have also been investigated for their roles in treatment monitoring and relapse prediction. Compared with imaging-based assessments, which often lag behind underlying biological changes, liquid biopsy approaches enable dynamic tracking of tumor cell states, providing more timely information for evaluating treatment response.

At the level of CTCs, multiple clinical studies have demonstrated that changes in CTC counts during treatment are closely associated with therapeutic response and clinical outcomes. Across chemotherapy, endocrine therapy, and selected targeted therapies, early declines in CTC numbers are indicative of favorable response, whereas persistently elevated levels or subsequent increases are associated with therapeutic resistance and disease progression [74]. However, CTC dynamics primarily reflect overall disease activity rather than sensitivity to specific treatment regimens, limiting their predictive value for individual therapeutic benefit.

Beyond enumeration, molecular characterization of CTCs provides a more refined layer of information. Alterations in therapy-relevant targets or resistance-associated markers may reflect tumor cell adaptation under treatment pressure and help identify emerging resistance [80], [81]. However, current evidence is largely derived from tumor-specific or small cohorts, and lack of methodological standardization constrains broader clinical application.

In contrast, DTC-based assessment is more closely aligned with long-term relapse risk. DTCs can remain detectable in the bone marrow despite surgery and/or adjuvant therapy, and their persistence is significantly associated with subsequent disease recurrence [82]. These findings support the use of DTCs as indicators of minimal residual disease (MRD) and suggest that they may reflect the extent of biological disease control following treatment.

From a temporal perspective, CTCs and DTCs capture distinct but complementary phases of treatment response. CTCs reflect short-term changes in dissemination dynamics and treatment response, whereas DTCs represent the long-term reservoir of residual disease and relapse potential.

Overall, CTC/DTC analyses are unlikely to function as standalone decision-making tools but may complement imaging and clinical parameters. Their integration may enable multidimensional assessment frameworks that bridge immediate treatment response with long-term relapse risk, thereby supporting more precise and time-resolved clinical management strategies.

## 4 Emerging technologies, research strategies, and future directions

Despite substantial advances in circulating tumor cell (CTC) and disseminated tumor cell (DTC) research, key methodological challenges remain in defining their biological roles and clinical utility in bone metastasis. In particular, reliably extracting informative signals from highly heterogeneous, dynamically evolving, and extremely rare cell populations remains a central bottleneck.

From a research paradigm perspective, conventional bulk-level analyses are insufficient to resolve the functional diversity of CTCs and DTCs. Although pronounced genetic, epigenetic, and phenotypic heterogeneity exists within these populations, only a small fraction of cells exhibits true bone tropism and metastatic competence. Moreover, DTCs in the bone marrow occupy dynamic and context-dependent states, with dormancy and reactivation tightly regulated by microenvironmental cues. These features necessitate a shift from binary detection (“presence or absence”) toward a more refined framework addressing which cells, in which states, and at what time points exert functional relevance.

Against this backdrop, emerging strategies—including single-cell multi-omics analyses, spatial omics, functional model systems, and multimodal data integration—provide critical tools to overcome current limitations. These approaches enable high-resolution dissection of CTC/DTC heterogeneity and their interactions with the bone microenvironment, while supporting the development of more predictive risk stratification models and identification of actionable intervention targets. This section highlights advances across four interconnected dimensions—single-cell and multi-omics profiling, spatial mapping, functional validation models, and integrative data frameworks—and discusses their translational potential and current limitations in the context of precision management of bone metastasis.

### 4.1 Single-cell and multi-omics strategies for dissecting CTC/DTC heterogeneity

A defining biological feature of CTCs and DTCs in bone metastasis is their pronounced intercellular heterogeneity, which is often obscured in bulk-level analyses. Accumulating evidence indicates that only a minority of subpopulations possess genuine bone tropism, long-term survival capacity, or reactivation potential, underscoring the necessity of single-cell–level characterization for understanding the metastatic cascade. Importantly, CTC/DTC heterogeneity should not be interpreted merely as molecular variability, but as functional diversity across the metastatic timeline. At the level of CTCs, single-cell analyses may distinguish epithelial-like, mesenchymal-like, hybrid EMT, clustered, immune-evasive, and bone-homing competent states. These states differ in their capacity for survival under shear stress, immune evasion, endothelial arrest, chemokine sensing, and extravasation into the bone marrow. For example, CTC clusters or blood cell-associated CTCs may exhibit enhanced survival and metastatic efficiency, whereas CTCs enriched for chemokine receptors and adhesion programs may be more competent for bone-directed homing. At the DTC level, heterogeneity is reflected by the coexistence of dormant, immune-evasive, metabolically adapted, and reactivation-competent states within the bone marrow niche. Dormant DTCs are generally characterized by slow-cycling or quiescent programs, stress adaptation, and niche dependence, whereas reactivation-competent DTCs may display proliferative, inflammatory, metabolic, or mechanotransductive programs that support escape from dormancy. Therefore, the functional relevance of single-cell heterogeneity lies in linking cellular states to specific steps of the metastatic cascade, including circulation survival, bone marrow seeding, niche integration, dormancy maintenance, and metastatic outgrowth.

This perspective also emphasizes that no single marker is likely to define metastatic competence. Instead, bone metastatic potential may be better inferred from coordinated transcriptional or multi-omic programs that integrate epithelial–mesenchymal plasticity, immune interaction, chemokine responsiveness, adhesion capacity, cell-cycle state, and metabolic adaptation. Accordingly, single-cell approaches should be interpreted together with functional validation models and longitudinal clinical outcome data to distinguish biologically relevant CTC/DTC states from transient or platform-dependent molecular variation.

The application of single-cell transcriptomic sequencing has enabled systematic and unbiased profiling of transcriptional heterogeneity within CTC/DTC populations [83], [84]. Recent studies have shown that distinct CTC subpopulations differ substantially in chemokine receptor expression, adhesion molecule profiles, stress response pathways, and metabolic states, with specific subsets exhibiting transcriptional features associated with bone tropism and metastatic fitness [28], [83]. Similarly, single-cell analyses of bone marrow DTCs have revealed substantial heterogeneity and identified dormancy-associated pathways, supporting the view that disseminated cells may occupy multiple functional states rather than a strictly binary dormant/proliferative pattern [84].

Building on these findings, single-cell multi-omics approaches have further expanded the resolution of functional state analysis. By integrating transcriptomic, chromatin accessibility, DNA methylation, and proteomic information, these strategies provide a more comprehensive framework for interrogating genetic background, epigenetic regulation, and phenotypic output [85]. Compared with single-modality data, such multidimensional integration is more effective in identifying functionally relevant cell subsets and potential regulatory nodes governing metastatic behavior.

However, several technical and interpretative challenges remain. The extremely low abundance of CTCs and DTCs introduces substantial risk of selection bias during enrichment and amplification, potentially leading to systematic underrepresentation of specific subpopulations. In addition, the complexity of single-cell datasets complicates data interpretation, as transcriptional states do not always directly correspond to functional phenotypes. Furthermore, cross-sectional sampling provides limited insight into temporal state transitions, particularly in the context of dormancy and reactivation.

Accordingly, the value of single-cell and multi-omics approaches lies not in generating increasingly large datasets, but in their integration with longitudinal sampling, functional validation models, and clinical outcome data. Within such a framework, high-resolution molecular profiles can be linked to defined cellular states and temporal contexts, thereby enabling biologically interpretable and clinically relevant insights into bone metastasis.

### 4.2 Spatial omics for resolving CTC/DTC–niche interactions

Although single-cell sequencing has greatly improved the molecular resolution of CTC/DTC heterogeneity, it generally requires tissue dissociation and therefore loses the spatial context in which DTCs interact with osteoblasts, endothelial cells, osteoclasts, stromal cells, and immune components. This limitation is particularly relevant for bone metastasis, because DTC fate is strongly influenced by spatial proximity to endovascular, endosteal/osteoblastic, osteoclast-enriched, and immune-regulatory niches [44], [49], [56], [64].

Spatial transcriptomics, spatial proteomics, multiplex immunofluorescence, and imaging mass cytometry provide complementary tools for mapping tumor cells and niche components within preserved tissue architecture [86], [87], [88]. By integrating gene or protein expression with anatomical localization, these approaches can identify whether dormant or reactivation-prone tumor cells are preferentially positioned near specific vascular structures, osteoblast-lineage cells, osteoclast-rich remodeling areas, macrophage-enriched regions, or immunosuppressive cell clusters. Such spatial information may help define dormancy-supportive and outgrowth-permissive “neighborhoods” that cannot be resolved by dissociated single-cell profiling alone.

In the context of a CTC/DTC-centered temporal framework, spatial omics may be particularly useful for linking cellular state to niche position. For example, spatially resolved analyses could clarify whether DTCs with quiescent transcriptional programs are enriched near stable vascular or osteoblastic niches, whereas proliferative or inflammatory DTC states are associated with angiogenic remodeling, osteoclast activation, or macrophage-dominated microenvironments. When combined with single-cell transcriptomics, lineage tracing, and functional perturbation models, spatial omics may therefore help distinguish causal niche interactions from simple cell-state associations.

However, several limitations should be considered. Bone tissue poses technical challenges for spatial omics because mineralized matrix, decalcification procedures, tissue autofluorescence, and low DTC abundance can compromise RNA/protein preservation and detection sensitivity. In addition, most spatial studies remain cross-sectional and cannot directly reconstruct the long latency between DTC seeding, dormancy, and metastatic relapse. Therefore, future studies should integrate spatial omics with longitudinal sampling, bone-mimetic models, and clinical outcome data to determine whether spatially defined CTC/DTC–niche interactions can predict dormancy escape or guide therapeutic intervention.

### 4.3 Functional models and biomimetic bone microenvironments: From association to causality

Although single-cell and multi-omics strategies have substantially improved the resolution of CTC/DTC heterogeneity, correlation-based data alone are insufficient to resolve key causal questions in bone metastasis, such as which molecular features directly determine bone homing, dormancy, or reactivation capacity. Accordingly, experimental systems that enable direct functional interrogation of CTC/DTC behavior under controlled conditions are essential for validating mechanistic hypotheses and identifying actionable targets.

Conventional mouse models remain indispensable for studying bone metastasis, particularly for evaluating long-term tumor cell behavior within intact immune systems and physiologically relevant bone remodeling contexts. However, these models typically rely on the introduction of large numbers of tumor cells, limiting their ability to faithfully recapitulate the low-abundance state of CTCs and DTCs observed clinically and to resolve rare events such as dormancy and reactivation. In addition, species-specific differences and substantial experimental time requirements restrict their utility for high-throughput mechanistic screening.

To overcome these limitations, in vitro functional models have been increasingly incorporated into CTC/DTC research. Three-dimensional co-culture systems and microfluidic platforms can partially reconstruct bone marrow vascular architecture, fluid shear stress, and cell–cell interactions, thereby providing controlled environments for investigating CTC adhesion, extravasation, and early colonization. A key advantage of these systems lies in their capacity for real-time tracking of single-cell behavior and precise perturbation of specific signaling pathways, enabling direct causal testing of their roles in bone homing and dormancy regulation [89].

More recently, biomimetic bone microenvironment models have further strengthened the transition from association to causality. By incorporating osteoblasts, osteoclasts, bone marrow stromal cells, and mineralized matrix components, these systems partially reconstitute the physical and biochemical features of bone tissue in vitro, allowing systematic evaluation of DTC survival, dormancy, and reactivation under defined microenvironmental conditions [90]. Compared with conventional two-dimensional cultures, such models more effectively capture the active and instructive role of the microenvironment in shaping DTC fate.

Nevertheless, current biomimetic systems remain limited in fully reproducing the dynamic complexity of the bone microenvironment, particularly with respect to immune components and long-term bone remodeling processes. The lack of standardized platforms further complicates cross-study comparability. Accordingly, these models should be regarded primarily as tools for mechanistic validation and hypothesis testing, rather than substitutes for in vivo systems or clinical investigation.

Taken together, the value of functional models and biomimetic bone microenvironments lies in their complementarity with single-cell multi-omics approaches. While high-resolution profiling identifies candidate cell states and regulatory pathways, functional systems provide the necessary framework to determine whether these candidates exert causal effects. Integration of functional modeling with longitudinal sampling and clinical outcome data will be critical for bridging the gap between molecular discovery and clinically actionable intervention in bone metastasis. However, translating these experimentally validated mechanisms into clinically applicable strategies remains a major challenge.

### 4.4 Challenges and perspectives: From research to translational application

Despite substantial technological and mechanistic advances in CTC and DTC research, significant barriers remain before their integration into bone metastasis management can be achieved. The extreme rarity and heterogeneity of CTCs and DTCs generate substantial variability across studies in detection rates, molecular characterization, and clinical correlations, thereby limiting reproducibility and comparability. The lack of standardized protocols for sample processing, analytical platforms, and data interpretation continues to impede clinical translation.

A second limitation lies in the predominance of correlative findings. Although single-cell and multi-omics approaches have delineated candidate cell states and regulatory pathways, relatively few mechanisms have been validated through robust functional interrogation across complementary model systems. This gap is particularly evident in studies of dormancy and reactivation, which are inherently time-dependent processes that cannot be adequately resolved by cross-sectional sampling alone. Without longitudinal integration of biological and clinical data, mechanistic insights remain difficult to operationalize. In addition, the prolonged and highly variable duration of metastatic dormancy creates a major feasibility challenge for clinical studies. In some tumor types, particularly breast cancer, dormant DTCs may persist for many years before relapse occurs, making it difficult to define appropriate sampling intervals, follow-up duration, and clinically meaningful endpoints in prospective human studies. This long latency also complicates the direct validation of CTC/DTC-based predictors of dormancy escape and metastatic reactivation [44], [45], [79], [82].

From a clinical standpoint, CTCs and DTCs are currently better positioned as instruments for risk stratification and prognostic assessment than as standalone decision-making tools. Their greatest potential may lie in complementing imaging, molecular subtyping, and established clinical parameters within multidimensional decision frameworks. In bone metastasis management, a central challenge is to define which patient subsets derive tangible benefit from CTC/DTC-informed early intervention, thereby balancing earlier detection against the risk of overtreatment.

Future progress will depend on several coordinated priorities. First, integration of longitudinal single-cell profiling with functional validation models may enable more precise identification of actionable cellular states governing bone homing, dormancy, and reactivation. Second, development of standardized detection workflows and interpretive criteria is essential to enhance cross-study comparability and support multicenter validation. Third, incorporation of CTC/DTC endpoints into prospective clinical trial designs will be critical to determine their value in treatment adaptation, relapse prediction, and dynamic risk assessment.

Taken together, CTC/DTC research in bone metastasis is moving from descriptive detection toward structured translational application. Future progress will require aligning high-resolution biological insights with standardized workflows, functional validation, longitudinal clinical data, and clearly defined decision points.

## 5 Conclusion

Bone metastasis should not be conceptualized as a late-stage event defined by radiographically detectable lesions, but rather as a time-structured, evolutionarily constrained process that unfolds long before clinical manifestation. In this framework, metastatic progression is governed not simply by dissemination efficiency, but by the capacity of tumor cells to persist, adapt, and transition between functional states within the bone marrow microenvironment. The bone marrow thus emerges not merely as a passive reservoir, but as an active regulatory hub that imposes selective pressures and orchestrates the survival, dormancy, and reactivation of disseminated tumor cells (DTCs). Bone-specific remodeling dynamics, including osteoblast–osteoclast coupling, further provide a uniquely permissive yet tightly regulated context for metastatic latency and abrupt outgrowth.

Within this time-resolved model, circulating tumor cells (CTCs) and DTCs represent complementary but non-redundant readouts of metastatic evolution. Rather than serving solely as biomarkers, they provide a conceptual framework for linking early dissemination dynamics with long-term disease reservoirs. This perspective shifts clinical interpretation from a static, lesion-centered paradigm toward a dynamic, biology-informed understanding of metastatic risk, in which dissemination, dormancy, and reactivation are viewed as temporally distinct but mechanistically connected phases.

A critical implication of this framework is that the clinically relevant question is not simply whether metastasis will occur, but when and under what conditions dormant disease transitions into overt progression. Defining this dormancy–reactivation window—particularly in the context of evolving bone remodeling states—represents a central challenge for the field. Importantly, CTC/DTC-informed approaches are not intended to replace existing clinical tools, but to contextualize uncertainty and refine decision-making by identifying biologically meaningful time points for intervention.

Looking forward, integration of single-cell multi-omics, spatial omics, functional modeling, and biomimetic microenvironment systems will be essential for resolving the cellular states, spatial niche interactions, and regulatory circuits that govern metastatic competence.

Taken together, a CTC/DTC-centered framework redefines bone metastasis from a detectable endpoint to a temporally evolving process. By aligning mechanistic insight with temporal resolution, this perspective provides a conceptual foundation for transitioning from reactive treatment of established metastases toward proactive, biology-guided intervention strategies.

## CRediT authorship contribution statement

**Zijie Yuan:** Writing – original draft, Investigation, Conceptualization. **Hao Zhang:** Writing – review & editing, Investigation. **Jingyu Xing:** Writing – review & editing, Investigation. **Xinghao Cao:** Writing – review & editing, Visualization, Data curation. **Genkun Liu:** Writing – review & editing, Investigation. **Tao Tan:** Writing – review & editing, Investigation. **Chenglong Zhao:** Writing – review & editing, Supervision. **Cheng Yang:** Writing – review & editing, Supervision.

## Funding

This work was supported by the 10.13039/501100001809National Natural Science Foundation of China (82372910). The funding source had no role in the design of the review, interpretation of the literature, writing of the manuscript, or the decision to submit the article for publication.

## Declaration of competing interest

The authors declare that they have no known competing financial interests or personal relationships that could have appeared to influence the work reported in this paper.
